# Filovirus Virulence in Interferon α/β and γ Double Knockout Mice, and Treatment with Favipiravir

**DOI:** 10.3390/v11020137

**Published:** 2019-02-03

**Authors:** Jason E. Comer, Olivier Escaffre, Natasha Neef, Trevor Brasel, Terry L. Juelich, Jennifer K. Smith, Jeanon Smith, Birte Kalveram, David D. Perez, Shane Massey, Lihong Zhang, Alexander N. Freiberg

**Affiliations:** 1 Department of Microbiology and Immunology, University of Texas Medical Branch at Galveston, Galveston, TX 77555, USA; trbrasel@utmb.edu; 2 Office of Regulated Nonclinical Studies, University of Texas Medical Branch at Galveston, Galveston, TX 77555, USA; jensmit1@utmb.edu (J.S.); chmassey@utmb.edu (S.M.); 3 Sealy Institute for Vaccine Science, University of Texas Medical Branch at Galveston, Galveston, TX 77555, USA; anfreibe@utmb.edu; 4 The Center for Biodefense and Emerging Infectious Diseases, University of Texas Medical Branch at Galveston, Galveston, TX 77555, USA; 5 Department of Pathology, University of Texas Medical Branch at Galveston, Galveston, TX 77555, USA; olescaff@utmb.edu (O.E.); tljuelic@utmb.edu (T.L.J.); jeksmith@UTMB.EDU (J.K.S.); bkkalver@utmb.edu (B.K.); dadperez@tamu.edu (D.D.P.); lihzhang@utmb.edu (L.Z.); 6 Experimental Pathology Laboratories, Inc., Sterling, VA 20167, USA; nneef@7thwavelabs.com; 7 Institute for Human Infections and Immunity, University of Texas Medical Branch at Galveston, Galveston, TX 77555, USA

**Keywords:** filovirus, Ebola virus, mouse, interferon receptor knockout

## Abstract

The 2014 Ebolavirus outbreak in West Africa highlighted the need for vaccines and therapeutics to prevent and treat filovirus infections. A well-characterized small animal model that is susceptible to wild-type filoviruses would facilitate the screening of anti-filovirus agents. To that end, we characterized knockout mice lacking α/β and γ interferon receptors (IFNAGR KO) as a model for wild-type filovirus infection. Intraperitoneal challenge of IFNAGR KO mice with several known human pathogenic species from the genus Ebolavirus and Marburgvirus, except Bundibugyo ebolavirus and Taï Forest ebolavirus, caused variable mortality rate. Further characterization of the prototype Ebola virus Kikwit isolate infection in this KO mouse model showed 100% lethality down to a dilution equivalent to 1.0 × 10^−1^ pfu with all deaths occurring between 7 and 9 days post-challenge. Viral RNA was detectable in serum after challenge with 1.0 × 10^2^ pfu as early as one day after infection. Changes in hematology and serum chemistry became pronounced as the disease progressed and mirrored the histological changes in the spleen and liver that were also consistent with those described for patients with Ebola virus disease. In a proof-of-principle study, treatment of Ebola virus infected IFNAGR KO mice with favipiravir resulted in 83% protection. Taken together, the data suggest that IFNAGR KO mice may be a useful model for early screening of anti-filovirus medical countermeasures.

## 1. Introduction

Filoviruses (family *Filoviridae*) are non-segmented, negative-sense RNA viruses responsible for causing several outbreaks of hemorrhagic fever throughout Africa. Five species are classified in the genus level of *Ebolavirus*, including *Zaire ebolavirus*, *Sudan ebolavirus*, *Bundibugyo ebolavirus, Taï Forest ebolavirus,* and *Reston ebolavirus*. *Marburg Marburgvirus* is the only known species of genus *Marburgvirus* [1] and only include Marburg and Ravn virus. Ebola virus (EBOV) and Marburg virus (MARV) are associated with high fatality rates ranging from 25% to 90% [2,3,4]. Sudan virus (SUDV) and Bundibugyo virus (BDBV), also cause severe disease but have so far been responsible for fewer and smaller outbreaks. Taï Forest virus (TAFV) has only caused a single known case, which did not result in fatality and Reston virus (RESTV), and is deemed nonpathogenic for humans [5,6]. EBOV is transmitted when individuals come in contact with infectious virus through abrasions in the skin, exposure of their mucosal tissues, or parental exposure [7]. The incubation period varies and reasoned to be between 2 and 21 days with an average incubation period of 7–10 days. The disease usually presents with flu-like symptoms accompanied by gastro-intestinal symptoms and as it progresses maculo-papulary rash, petichae, conjunctival hemorrhage, epistaxis, melena, hematemesis, shock, and encephalopathy can develop [8]. 

Multiple animal models have been developed to study filovirus-induced pathogenicity or evaluate vaccine candidates and antiviral treatment options. Non-human primates (NHPs) are considered as the “gold standard” model for studying filovirus pathogenesis because of the similarity in clinical disease as observed in patients [9]. However, it is not practical or ethnical to use NHPs in early screening studies for medical countermeasures [10]. Ferrets have recently been described as a novel model to study filovirus pathogenicity, since they recapitulate aspects of human disease when infected with EBOV, SUDV, and BDBV, but logistical issues including cost, viability, husbandry requirements, and availability of reagents make this model impractical for early testing [11,12,13,14]. Mouse-adapted EBOV and hamster-adapted MARV are lethal in Syrian hamsters and cause disease manifestations, similar to those of NHP and humans including coagulation abnormalities and unchecked immune responses [15,16], but availability of reagents makes again this model difficult to work with [17,18]. Until now, guinea pigs [19,20,21,22,23,24,25,26,27] and mice [28,29,30,31,32,33,34] have been the most widely used smaller animal models for preliminary testing. However, species adapted viruses are necessary to result in disease development in immune-competent animals. In the process of adaptation, viruses acquire numerous nucleotide/amino acid mutations that notably antagonize interferon response contributing to virulence in their rodent host [31,35], and could present a difficulty in testing virus-specific antiviral treatments. While a significant level of work has been conducted in the mouse model of filovirus disease, there is still need for further model development because wild-type filovirus can only infect immunocompromised mice as small rodent animals [36]. Previously, knockout (KO) mouse models that either lack expression of the cytoplasmic Signal Transducer and Activator of Transcription-1 (STAT-1) protein impairing response to IFN α/β, and γ stimulation or of IFN-α/β receptors have shown to uniformly succumb to Ebola virus using intraperitoneal route or aerosol exposure [17,28,29,32,37,38,39,40]. It is however interesting to note that STAT1 KO mouse model was recently demonstrated inappropriate for efficacy testing of a promising vector-based filovirus vaccine [41]. Other models such as the severe combined immunodeficiency (SCID) mouse model, which lacks B and T cell response, has also been shown to be susceptible to infection with wild-type Ebola virus [32], and progression of disease was generally delayed compared to the two aforementioned mouse models. 

Under the NIH/NIAID Animal Models of Infectious Diseases Contract we were tasked with characterizing small animal models of wild-type filovirus infections. Here we present the use of mice lacking α/β and γ interferon receptors (IFNAGR KO) as a new small animal model for wild-type filovirus infection. In addition to susceptibility and pathogenicity studies, we also set out to determine the usefulness of this KO mouse model for antiviral treatment with the previously described broad-spectrum antiviral favipiravir. 

## 2. Materials and Methods

### 2.1. Ethics Statement 

All animal experiments were approved by the Institutional Animal Care and Use Committee of The University of Texas Medical Branch (protocol number 1712017 approved on 01 December 2017) and performed following the guidelines of the Association for Assessment and Accreditation of Laboratory Animal Care International (AAALAC) by certified staff in an AAALAC-approved facility.

### 2.2. Cells and Viruses

Ebola virus (EBOV), variant Zaire Kikwit; Sudan virus (SUDV), variant Gulu; Bundibugyo virus (BDBV); Taï Forest virus (TAFV); and Marburg virus (MARV), isolate Angola. All virus inocula were tested for sterility, mycoplamsa, endotoxin, and were subjected to deep sequencing. All virus stocks were obtained from BEI Resources (Manassas, VA, USA). Virus titers were determined by plaque assay on low passage (< 20) Vero E6 cells (BEI Resources) as previously described [42]. All work with infectious virus was performed under Biosafety level 4 conditions in the Robert E. Shope, M.D., and the Galveston National Laboratory at The University of Texas Medical Branch (UTMB).

### 2.3. Animal Studies 

Male and female 8–18 week old interferon α/β and γ receptor double knockout (IFNAGR KO) mice on a C57BL/6J background were provided by Dr. Slobodan Paessler from a colony bred at UTMB. 

### 2.4. Mouse Challenge and Observations

Virus administration was performed via intraperitoneal (i.p.) injection (Study Day 0), and the viral dose was verified by standard plaque assay as previously described [42]. Each group was gender matched to the extent possible. Animals were monitored daily for development of clinical signs of disease and changes in body weight. Clinical scoring and health assessments were performed and documented at each observation. The following scoring chart was used: 1 – normal; 2 – ruffled fur, lethargic (twice daily observation 6–8 hours apart; 3 – score 2 criteria plus hunched posture, orbital tightening (three times daily observation, with the third observation occurring 4–6 hours after the second observation); 4 – score 3 criteria plus reluctance to move when stimulated, OR ≥20% weight loss (immediate euthanasia). Body weights were monitored daily through day 10 post-challenge then every 3rd day until the end of the study. Terminal bleeds were performed at time of euthanasia. 

### 2.5. Susceptibility Study

Groups of five mice were inoculated with 1.0 × 10^4^ plaque-forming units (pfu) of each virus via the i.p. route. Animals were monitored daily for development of clinical signs of disease.

### 2.6. Serial Dosing Study

Based on the susceptibility study, EBOV was selected for further model development. To determine the mean lethal dose (LD_50_), five groups of five IFNAGR KO mice were infected with 1.0 × 10^4^–1.0 × 10^0^ pfu via the i.p. route. In a separate experiment, six groups of six mice each were infected with 1.0 × 10^2^–1.0 × 10^−3^ pfu of EBOV. Monitoring was performed as described above. The LD_50_ with its 95% confidence interval was estimated by logit model using SAS 9.3 (SAS Institute Inc., Cary, NC, USA).

### 2.7. Natural History Study

To investigate disease development of EBOV infection in IFNAGR KO mice, animals were infected with 1.0 × 10^2^ pfu of EBOV via the i.p. route. Seven pre-selected mice (3 male/4 female or 4 male/3female) were euthanized per day from day 0 (prior to challenge) through day 6 post-challenge. Health monitoring was performed as previously described. At time of euthanasia, whole blood was collected by cardiac puncture and necropsies performed. Liver and spleen were collected and fixed in 10% formalin (Thermo Fisher Scientific) for histopathological analysis. Animals were not perfused prior to necropsy.

### 2.8. EBOV qRT-PCR

An amount of 50 µL of sera was added to 250 µL of TRIzol LS (Life Technologies, Carlsbad, CA, USA) and stored at ≤ −60 °C until used for RNA exaction. RNA was extracted from samples using the Zymo Research Direct-zol^™^ RNA MiniPrep kit (Zymo Research, Irvine, CA, USA). For sample quantification, each assay plate contained a standard curve prepared using EBOV glycoprotein gene synthetic RNA (1.0 × 10^3^ to 1.0 × 10^10^ genome equivalents/µL [GEq/µL] in duplicate wells). For the RT-PCR, QuantiFast Probe RT-PCR Master Mix and QuantiFast RT Mix (Qiagen, Ilden, Germany) were used in conjunction with Forward primer: 5′- TTT TCA ATC CTC AAC CgT AAg gC-3′, Reverse primer: 5′- Cag TCC ggT CCC AgA ATg Tg-3′ and Probe: 5′-6FAM- CAT gTg CCg CCC CAT CgC TgC-MGBNFQ-3′. Primers and probe targeted the glycoprotein (GP) gene from EBOV (GenBank accession no. AF086833). The RT-PCR was conducted on a Bio-Rad CFX96^TM^ Real-Time PCR 

### 2.9. Hematology and Serum Chemistry

Blood collected in the EDTA tubes was analyzed for hematology parameters using a HEMAVET Multispecies Hematology Analyzer (Drew Scientific, Miami Lakes, FL, USA). Blood collected in the lithium heparin tubes was analyzed for changes in clinical chemistry parameters using a VetScan VS2 Chemistry Analyzer and Comprehensive Diagnostic Profile rotators (Abaxis, Union City, CA, USA). 

### 2.10. Histology and Immunohistochemistry

Liver and spleen samples were harvested at necropsy, fixed in 10% neutral buffered formalin, and sent to Experimental Pathology Laboratories, Inc. (EPL^®^ Sterling, VA, USA) for processing. Briefly, the samples were trimmed, paraffin embedded, sectioned at 4–6 µm with a microtome (one spleen section and two liver sections/mouse), placed on glass slides, and stained with hematoxylin and eosin. All tissue sections were examined by a board-certified veterinary pathologist. The presence of EBOV antigens in tissues was also investigated by conventional immunohistochemistry (IHC) techniques on unstained sections using a rabbit anti-Zaire Ebola virus-like particles (VLP) polyclonal antibody (IBT Bioservices, Rockville, MD, USA) and a secondary HRP-conjugated goat anti-rabbit polyclonal antibody (Abcam, Cambridge, MA, USA) at a dilution of 1:50, or 1:100, respectively. Observations were made with an Evos XL core microscope at 10 or 20X magnification. Staining was graded: 0 = no staining, 1 = rare, 2 = frequent, 3 = diffuse.

### 2.11. Favipiravir Treatment

Groups of 6 mice (3 male and 3 female) were challenged with 1.0 × 10^2^ pfu of EBOV via i.p. injection and monitored as described above. Starting 1-hour post-challenge, mice were treated with 150 mg/kg of Favipiravir (AdooQ Bioscience, Irvine, CA, USA) in 0.5% methylcellulose (100 µL volume) twice daily for 8 days via the oral gavage route. 

## 3. Results

### 3.1. Susceptibility of IFNAGR KO Mice to Filoviruses

**Figure 1:**
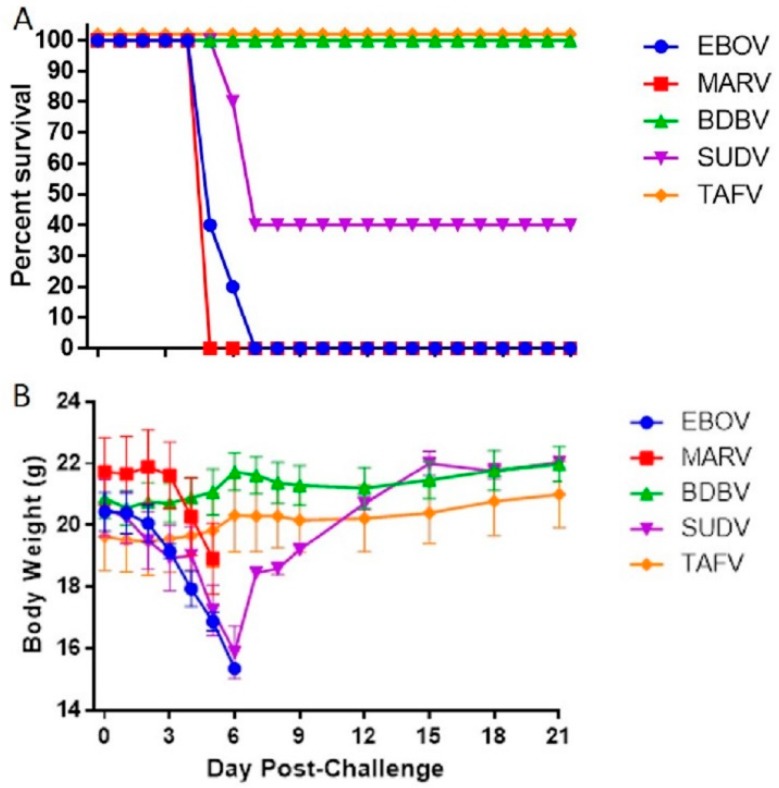
Screening of IFNAGR KO Mice for Susceptibility to Wild-Type Filoviruses. Groups of five mice were challenged with 1.0 × 10^4^ pfu of wild-type filovirus and observed survival (**A**) and body weight loss (**B**). The symbols represent mean group weights and the bars represent standard error.

In an initial susceptibility study, groups of 5 mice (2 male/3 female or 3 male/2 female) were challenged with 1.0 × 10^4^ pfu of virus via i.p. injection and morbidity and mortality observed for 21 days. All EBOV- and MARV-inoculated mice started respectively losing weight day 3 or 4, and reached euthanasia criteria between day 5 and 6 (Figure 1A). From challenge until death, EBOV and MARV-infected mice lost an average of 20% and 13% body weight, respectively (Figure 1B). Sixty percent (60%) of the mice challenged with SUDV died between day 6 and 7. Weight loss was recorded at day 3, with a reduction of 22.8% on average by day 6. The two surviving mice had clinical scores of 3 on days 5 to 10, then returned to normal (clinical score = 1, and no weight loss) by the end of the study at 21 days post infection. Finally, all mice challenged with BDBV or TAFV survived to the end of the study without any significant weight loss (Figure 1A,B), even though BDBV-challenged animals temporarily exhibited clinical scores of 2 on days 5 and 6. 

### 3.2. Determination of the Lethal Dose Range of EBOV in the IFNAGR KO Mouse Model 

**Figure 2:**
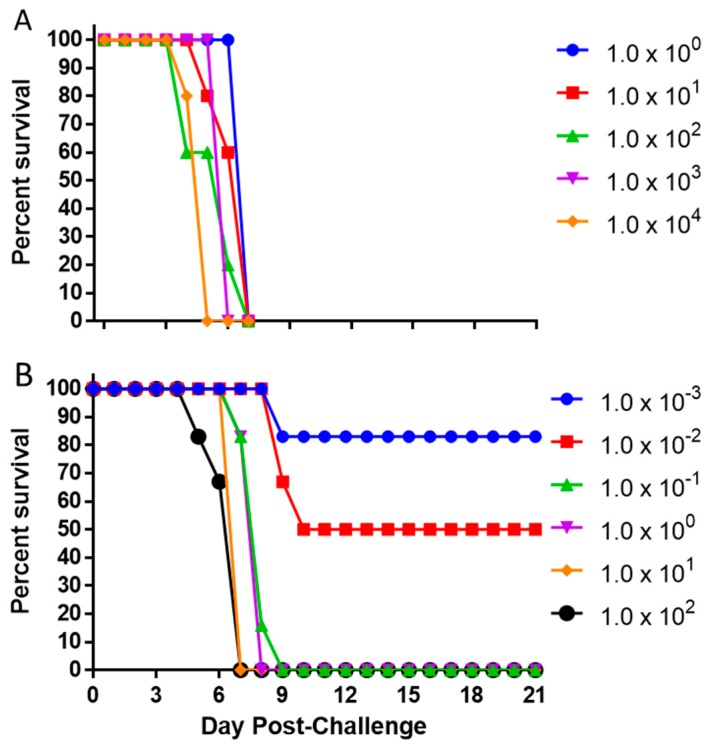
Survival after Challenge with Various Doses of EBOV. Mice (N = 5) were challenged with 1.0 × 10^4^–1.0 × 10^0^ pfu of EBOV (**A**). In a separate experiment, mice (N = 6) were challenged with 1.0 × 10^2^–1.0 × 10^−3^ pfu of EBOV (**B**).

Based on the results of the susceptibility study and the need for screening models for EBOV, we chose to further characterize EBOV in the IFNAGR KO mice. Groups of 5 mice (2 male/3 female or 3 male/2 female) were challenged with five serial 10-fold dilutions of EBOV starting at 1.0 × 10^4^ pfu. All tested challenge doses resulted in 100% mortality down to 1.0 × 10^0^ pfu (Figure 2A). The mice in the two highest dose groups, 1.0 × 10^4^ and 1.0 × 10^3^ pfu, respectively succumbed to infection by day 5 and 6, whereas animals inoculated with any lower dose died by day 7. To further characterize the lethality of EBOV in this model and to be able to determine a LD_50_ value, six additional groups of 6 mice each (3 male/3 female) were challenged with 10-fold serial dilutions of EBOV starting at 1.0 × 10^2^ pfu (Figure 2B). Here, EBOV was uniformly lethal down to 1.0 × 10^−1^ pfu with all deaths occurring between day 7 and 9. The 1.0 × 10^−2^ and 1.0 × 10^−3^ pfu doses resulted respectively in 50 and 17% lethality. The LD_50_ was estimated to be 0.007 PFU with the 95% confidence interval of 0.0007–0.0363, based on the dilutions of viral stock used. The limit of detection for our plaque assay using 6-well plates is 5 pfu/mL, therefore the LD_50_ was calculated based on target concentration of the dilution series and could not be verified by plaque assay. 

### 3.3. Progression of Ebola Virus Disease in the IFNAGR Mouse Model

**Figure 3:**
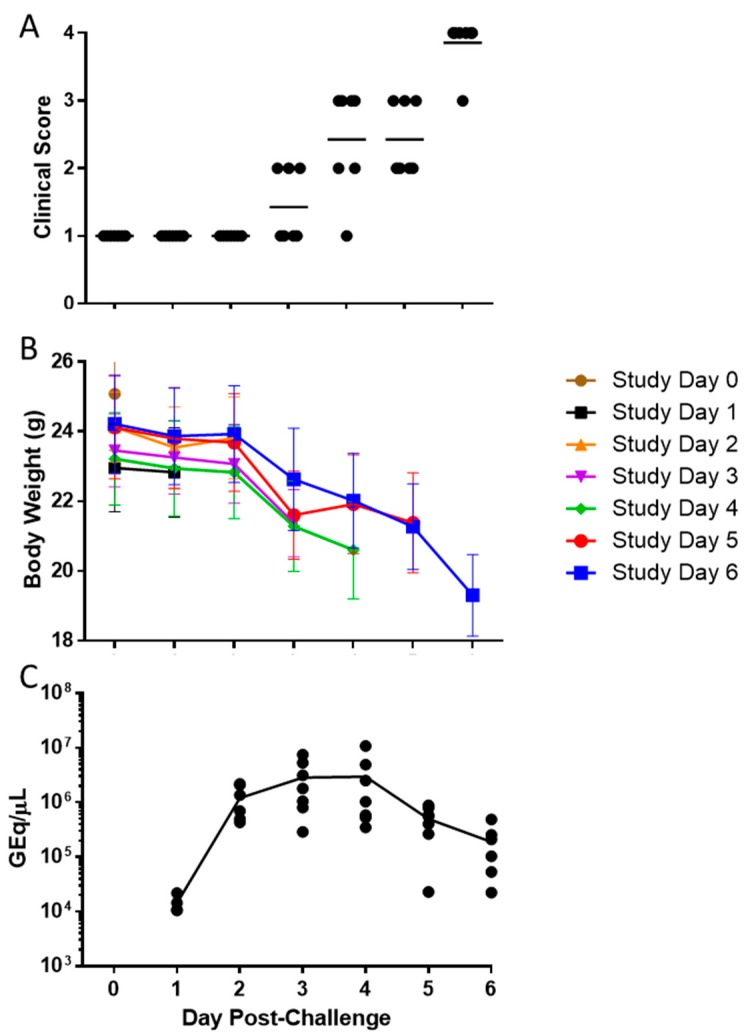
Clinical Scoring, Weight Loss, and Viremia after EBOV Challenge. Mice were challenged with 1.0 × 10^2^ pfu of EBOV and 7 mice were euthanized each from Day 0 through Day 6. Individual clinical scores (**A**) and body weight (**B**) were recorded over time with the horizontal bars indicating means. The levels of viral RNA in serum were also measured at the time of euthanasia and presented in genome equivalents per µL (GEq/µL; **C**).

To further characterize the pathogenicity of EBOV in IFNAGR KO mice, a natural history of disease study was performed. To ensure a uniform lethality, mice were challenged with a lethal dose of 1.0 × 10^2^ pfu of EBOV via i.p. injection and 7 pre-selected subjects were euthanized per day from day 0 (prior to challenge) through day 6 post-challenge. All mice scored a 1 from day 0 to 2 (Figure 3A). One of the seven animals (14%) euthanized on day 3 scored a 2 while all others were healthy. Body weight loss was observed starting between days 2 and 3, and clinical scores began to rise on day 4 and continued to rise though day 6 when all remaining animals were euthanized. Animals euthanized on day 6 lost an average of 20% of body weight from day 0 (Figure 3B). 

At the time of euthanasia, blood was collected and serum assayed for the level of EBOV RNA. The mean viral RNA detected increased from day 1 (1.43 × 10^4^ GEq/μL) to day 4 (2.96 × 10^6^ GEq/μL), concomitant of clinical symptoms onset and significant weight loss at day 3, and then began to decrease on day 5 (5.00 × 10^5^ GEq/μL) and 6 (1.89 × 10^5^ GEq/μL) (Figure 3C), at which time all animals exhibited a clinical score of 3 or 4.

### 3.4. Alteration of Hematologic and Liver Parameters During the Course of EBOV Infection in IFANGR KO Mice

**Figure 4:**
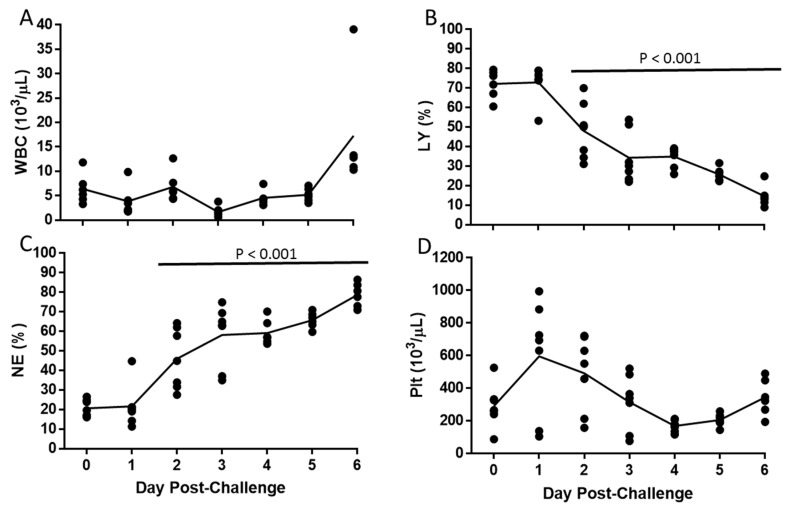
Hematological Changes following EBOV Challenge. Symbols (•) represent values for individual mice and the line represents means of White Blood Cell counts (WBC; **A**), Percent of lymphocytes (LY; **B**), Percent of neutrophils (NE; **C**), and Platelet counts (Plt; **D**). Horizontal bar indicates significant difference from Day 0.

The total white blood cell count remained relatively constant throughout the study (Figure 4A). However, there was a decrease in the percentage of lymphocytes and an increase in the percentage of neutrophils over the course of the study, starting day 2 (*p* < 0.001, Tukey–Kramer; Figure 4B,C). The mean number of platelets increased by day 1, and then decreased until day 4 (*p* > 0.05, Tukey–Kramer; Figure 4D). 

**Figure 5:**
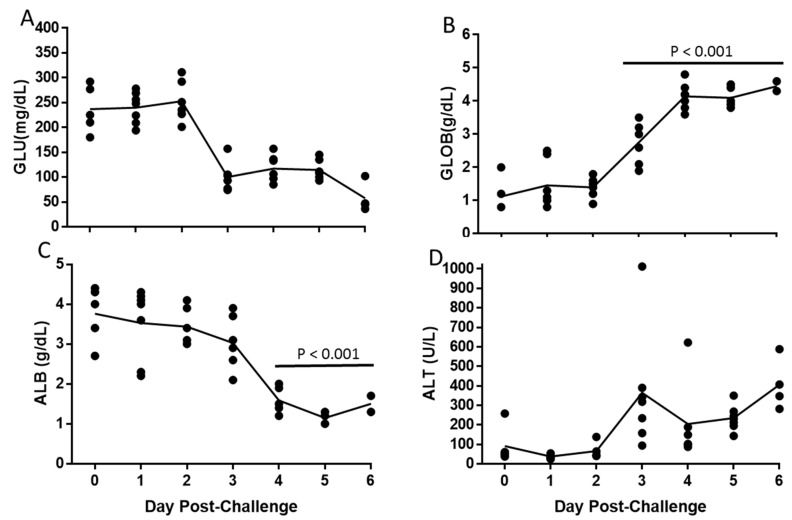
Changes in Serum Chemistry following EBOV Challenge. Symbols (•) represent values for individual mice and the line represents means of: Glucose (GLU; **A**), Globulin (GLOB; **B**), Albumin (ALB; **C**), and Alanine aminotransferase (ALT; **D**). Horizontal bar indicates significant difference from Day 0.

Glucose levels decreased between day 2 and 3 (*p* > 0.05, Tukey-Kramer, Figure 5A), which corresponded with the start of the loss of body weight. There was an increase in globulin levels starting day 3 (*p* < 0.001, Tukey–Kramer) and a decrease in albumin levels starting day 4 (*p* < 0.001, Tukey–Kramer; Figure 5B,C). There was also an increase in alanine aminotransferase (ALT) levels starting day 3 (*p* > 0.05, Tukey–Kramer, Figure 5D). The inversion of globulin and albumin levels and the increase in ALT were suggestive of liver damage, which was supported by histological evaluation. 

### 3.5. Histopathological Changes in EBOV-Infected IFNAGR KO Mice

**Figure 6:**
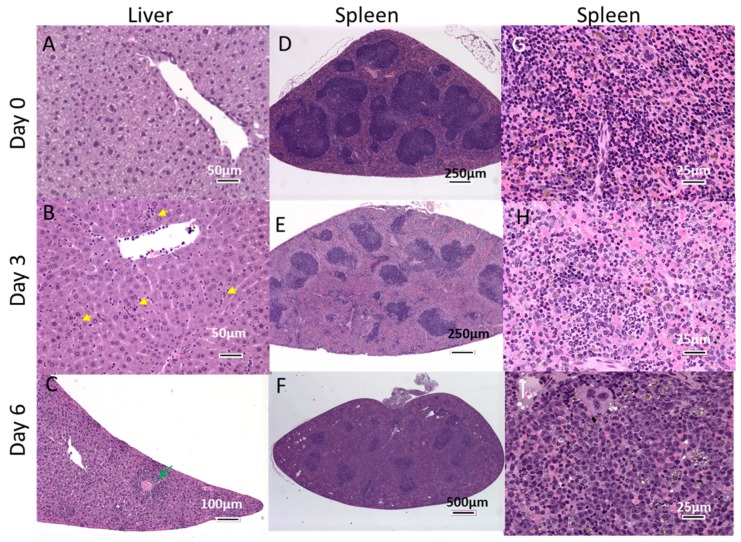
H&E Staining of Liver and Spleen following EBOV Challenge. Mice were challenged with 1.0 × 10^2^ pfu of EBOV and 6 mice were euthanized each day from Day 0 (unchallenged) through Day 6. Panels **A**, **B** and **C** show representative liver sections from mice euthanized on Days 0, 3 and 6 respectively. Representative spleen sections are shown in panels **D**, **E** and **F**. Panels **G**, **H** and **I** show the spleen sections at a higher magnification. Yellow arrowheads indicate focal sinusoidal inflammation in liver, green arrowhead shows perivascular inflammation.

The pathology observed in the liver and the spleen were broadly consistent with Ebola Virus Disease (EVD) described in human EBOV infections [43] and followed what has been reported in the NHP and other rodent models [15,20,30,38,44]. Histological changes in the liver appeared on day 2, and were characterized by occasional, randomly located small clusters of mixed inflammatory cells (neutrophils and monocytes) within the hepatic parenchyma that expanded the sinusoids locally. By day 3, the incidence and severity of hepatic inflammation was increased, and in a few cases, was accompanied by hypertrophy of sinusoidal lining cells compared to normal tissue (Figure 6A,B). Eosinophilic cytoplasmic inclusions were first identified at day 3 (Figure 6B). In general, inclusions were most easily identified within hepatocytes, but also occasionally within sinusoidal lining cells (endothelial or Kupffer cells). In more severe cases, inflammatory foci were distributed perivascularly around both portal areas and central veins, as well as randomly within the sinusoids, and comprised approximately equal proportions of neutrophils and macrophages (Figure 6C). Occasional single-cell necrosis of hepatocytes, mainly associated with inflammatory foci, was present in the more severely affected animals. In animals euthanized on day 4 to 6, there was an increase in the severity of inflammatory changes with a modest reduction in the neutrophil component in favor of a more florid macrophage presence. Hypertrophy of sinusoidal lining cells was also more prominent and the number of inclusion bodies increased. 

In the spleen, changes associated with viral challenge generally appeared from day 3 (Figure 6). Decreased lymphocytes were noted in the white pulp, with severity increasing with the progression of disease (Figure 6D–F). Note that splenic white pulp in the unchallenged animals of this particular mouse strain appeared to have homogenous, expanded T-cell areas, with no apparent follicle formation compared with typical wild-type mice. Diffuse histiocytosis (with increased brown pigment within macrophages in some cases), was also noted in challenged animals, and increased in severity with time Figure 6G–I). In contrast, diffuse neutrophil infiltration of the red pulp was most severe on day 3 and became less prominent with time (Figure 6H,I). This paralleled the reduction in the neutrophil component of hepatic inflammation over the course of the study.

**Figure 7:**
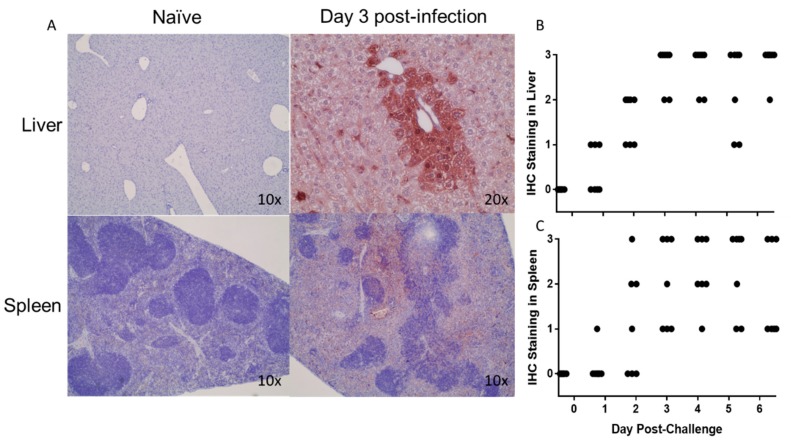
IHC Staining of Liver and Spleen. Virus antigen in liver and spleen samples were labeled with a rabbit anti-Zaire Ebola VLP polyclonal antibody. Virus antigen specific staining is was compared at different time points (**A**). The incidence of viral straining over time was measured in the liver (**B**) and spleen (**C**) using the following scale: 0 = no staining, 1 = rare, 2 = frequent, 3 = diffuse.

Presence of EBOV antigen was investigated in liver and spleen by IHC (Figure 7A). Virus antigen was detected in the liver of 3 of the 7 mice by day 1, and in all subsequent collected liver specimens, whose antigen staining was graded frequent to diffuse, starting day 2 (Figure 7B). Virus antigen in the spleen was overall detected later than in the liver as only 1 spleen was positive on day 1, 4 on day 2, and in all subsequent collected specimens starting day 3, time at which antigen staining levels became frequent to diffuse (Figure 7C). 

### 3.6. EBOV-Challenged IFNAGR KO Mice as a Screen Model for Antivirals 

**Figure 8:**
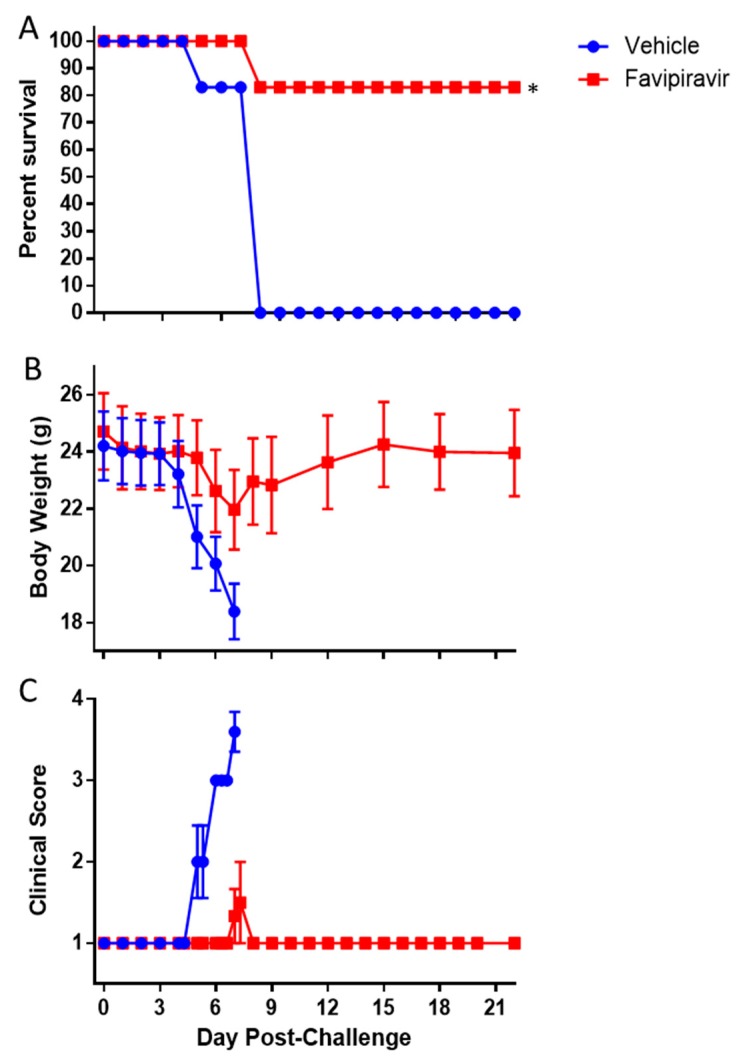
Efficacy of Favipiravir against EBOV in IFNAGR mice. Mice were challenged with 1.0 × 10^2^ pfu of EBOV then treated with 150 mg/kg of favipiravir twice daily for 8 days. The mice were observed for survival (**A**), body weight loss (**B**), and clinical score (**C**). The asterisk indicates statistical significance (*p* = 0.015, Fisher’s Exact Test). The bars indicate standard error.

To determine the usefulness of IFNAGR KO mice as a screening model for anti-filovirus countermeasures, we treated challenged mice with the antiviral Favipiravir. Indeed, Favipiravir was previously shown to be effective against wild-type EBOV in IFN-α/β receptor KO mouse models [45,46,47], mouse-adapted EBOV and MARV in respectively C57BL/6 or BALB/c mouse model [47,48,49], guinea pig-adapted SUDV in the guinea pig model [50] and wild-type EBOV or MARV in the NHP models [51,52]. Here, Favipiravir treatment was initiated 1 hour post-infection and infected mice were treated twice daily for 8 days. In the IFNAGR KO mouse model, favipiravir protected 83% of mice (5/6), while all vehicle-treated animals reached euthanasia criteria by day 7 (*p* = 0.015, Fishers Exact Test; Figure 8A). The non-survivor favipiravir-treated subject had an 18.4% reduction in body weight and a clinical score of 4 by day 7, similar to those in the vehicle-inoculated group (Figure 8B,C). The 5 surviving animals only showed a 9.9% (±1.4%) and 5.4% (±3.6%) reduction in body weight on day 7 and on the day 22, respectively (Figure 8B), with no clinical signs of disease (Figure 8C). 

## 4. Discussion

Availability of vaccines and therapeutics for prevention and treatment of filovirus infections would provide significant health benefits to communities and health workers in endemic areas. Products in later stages of development require the use of NHP efficacy testing as it is the system that most closely models human infection. However, a screening model is needed for products early in development as it is not feasible or ethical to perform efficacy screening in NHPs. Small animal models of filovirus infection have been developed, including guinea pigs, hamsters, and immunocompetent mice [15,20,28,40,53]. However, all of these models require adaption of the virus to establish infection, which reflects mutations leading to some viral proteins antagonizing interferon response of the animal model [31,54,55]. Thus, there is a need to screen anti-filovirus countermeasures against wild-type filoviruses to ensure the product be efficacious against the wild-type viruses that would be encountered in outbreak scenarios. Therefore, much research has focused on developing immunocompromised mice [28,29,30,32,45,46,47] and hamsters [56] to study wild-type filovirus infection. 

Here, the suitability of IFNAGR KO mice as small rodent model to study wild-type filovirus infection was initially investigated. While we showed the susceptibility of this model to EBOV, SUDV, and MARV as they caused clinical manifestations of disease and mortality, establishment of a small rodent model suitable for BDBV and TAFV will require further investigations. Wild-type BDBV did not cause clinical disease in the IFNAGR KO mice and wild-type SUDV was only 60% lethal, however both viruses are able to produce fatal disease in ferrets. Intranasal challenge with 1000 pfu of SUDV or BVBD results in 100% lethality [11] and intramuscular (i.m.) injection of 159 TCID_50_ of BDBV was also lethal and recapitulated many aspects of human disease, suggesting ferrets may be a more appropriate model [11,12]. The strains of BDBV and SUDV used here were the same as in the ferret work and deep sequencing confirmed that the consensus sequence agreed with the reference sequence for both viruses. Moreover, the stock of SUDV used in this work was fully lethal in a cynmologus macaque model (unpublished results). Taken together, this suggests that the viruses have not lost virulence and that the IFNAGR mice are less sensitive to infection than ferrets. 

We then focused on EBOV as this is the most characterized species in the immuno-deprived mouse models and there is a current need to screen anti-EBOV compounds in a small animal model. Bray et al. showed that 1 × 10^3^ pfu of EBOV given intraperitoneally (i.p.) was 100% lethal in 129 mice lacking Interferon (IFN)-α/β receptor [29]. This was confirmed by Lever et al. (2012), who went on to demonstrate that EBOV was fully virulent in this strain via the aerosol route as well [57]. The IFN α/β receptor knockout (KO) mouse model was further characterized by another group who determined it was less susceptible to EBOV than the previous studies [38]. In fact, a challenge dose of 5.0 × 10^3^ pfu by i.p. only resulted in 20% lethality. However, this strain was on a C57BL/6 background, which may explain the increased resistance as Haddock and colleagues recently showed that C57BL/6 mice have increased resistance to mouse-adapted EBOV compared to 129 mice [33]. However, Oestereich et al. showed that 1.0 × 10^3^ pfu of wild-type EBOV was fully lethal in IFN α/β receptor KO mice on a C57BL/6 background but only resulted in 20% mortality in mice with a 129/Sv background [47]. Further research is required to determine the effect of the genetic background on strain susceptibility. In the present study, we show that a dose of 1.0 × 10^−1^ pfu of wild-type EBOV is 100% lethal in the IFN-α/β, and γ receptor (IFNAGR) KO mouse model, which is also on the C57BL/6 background suggesting the loss of both Type I and Type II interferon signaling increases sensitivity to wild-type EBOV infection. Although wild-type EBOV infection in IFN-γ receptor KO mice has never been tested, our data are also in line with the fact that IFN-γ treatment of C57BL/6 IFN γ receptor KO mouse macrophages or of BALB/c IFN α/β receptor KO mice inhibited virus infection by EBOV GP/rVSV [58]. The fact that less than 1 pfu was fatal in this model is similar to what has been observed in NHPs. Alfson et al. demonstrated that a target dose of 1.0 × 10^−2^ of wild-type EBOV administered via i.m. injection was fully lethal in cynomolgus macaques [59]. This suggests that while plaques at not detectable in a cell culture the inoculum still contains infectious virus particles able to cause disease. 

EBOV-infected IFNAGR KO mice became neutrophilic and lymphopenic after challenge, which is in line with observations from EBOV-infected NHPs, guinea pigs, and other mouse strains [15,21,38,44,60,61,62]. The main histopathological features in the liver and spleen in this mouse model were also broadly consistent with what was previously described in the corresponding organs in Ebola patients [43], but also in NHPs, guinea pigs, and IFN α/β receptor KO mice [21,38,44]. Specifically, changes in the liver consisted of sinusoidal/perivascular macrophage and neutrophil inflammation associated with relatively minor single-cell hepatocyte necrosis that correlated with modest elevations in ALT. In the spleen, changes were characterized by histiocytosis and neutrophilia, accompanied by reductions in white pulp lymphoid tissue, which respectively reflected a decreased and increased circulating lymphocyte or neutrophil counts. 

Both immunocompromised and wild type mouse models have proved their usefulness in antiviral drug discovery. For example, favipiravir that selectively inhibits the RNA-dependent RNA polymerase [63] has been shown to provide protection to multiple mouse models, including IFN α/β KO [45], and NHPs challenged with EBOV or MARV [46,47,48,49,52]. As a proof of concept that IFNAGR KO mice may be used for initial screens of antivirals, we chose to treat EBOV-infected mice with favipiravir. Our results showed that favipiravir is also able to protect IFNAGR KO mice from a lethal wild-type EBOV challenge and support that this KO mouse model could be used for future evaluation of small molecule antiviral therapeutics. However, additional studies are required to determine the window of effectiveness of favipiravir in this model as was done in the IFN α/β receptor KO model which was shown to be 100% effective when treatment was initiated on day 6 post-challenge [47]. Altogether, our data suggest that the IFNAGR KO mouse model is another small animal model relevant to study wild-type EBOV pathogenesis and for screen for novel antivirals against wild-type filoviruses. The usefulness of this model in comparison to other immunocompromised mouse strains has yet to be determined, but the increased sensitivity does make it a more stringent model for product screening.
